# Revision of hip resurfacing arthroplasty with a bone-conserving short-stem implant: a case report and review of the literature

**DOI:** 10.1186/1752-1947-6-249

**Published:** 2012-08-20

**Authors:** Florian Schmidutz, Lorenz Wanke-Jellinek, Volkmar Jansson, Andreas Fottner, Farhad Mazoochian

**Affiliations:** 1 Department of Orthopedic Surgery, University of Munich (LMU), Campus Grosshadern, Marchioninistraße 15, Munich, 81377, Germany

**Keywords:** Arthroplasty, Bone conservation, Hip resurfacing, Metaphyseal, Metha®, Revision, Short stem

## Abstract

**Introduction:**

Suitable treatment of early failure of total hip replacement is critical in younger patients, as bone stock is lost and the functional outcome is impaired.

**Case presentation:**

We report the case of a 56-year-old Caucasian woman with early failure of hip resurfacing arthroplasty. While revision is usually performed with a conventional hip implant, this case report describes for the first time a revision procedure with a bone-conserving short-stem hip implant.

**Conclusions:**

Our approach allows further conservation of femoral bone stock and provides a long-term solution to the patient, which maintains the possibility of using a conventional hip implant should a second revision become necessary.

## Introduction

The good clinical results in hip arthroplasty have led to an increasing number of joint replacements in younger patients. Regardless, it is well known that this patient group faces an increased risk of early implant failure [1], which is probably related to their higher activity level. Revision surgeries often go along with loss of bone substance [2], resulting in more difficult procedures and an impaired functional outcome [3]. In order to facilitate potential revision surgeries, bone-preserving implants, such as hip resurfacing arthroplasty (HRA) and short-stem arthroplasty (SHA) implants, have been developed and recently have gained increasing popularity.

However, only little data is available how much bone stock is conserved, and moreover, if revision procedures are actually facilitated by the use of bone-preserving implants. So far, only a few studies have reported on the revision of failed HRA implants and all revisions have exclusively been performed by the use of a conventional stem [4,5]*.*

In this report, we describe a woman with early failure of HRA. Revision was performed with a bone-conserving short-stem hip implant, which minimizes the bone loss on the femoral side in order to facilitate potential revision surgery.

## Case presentation

A 56-year-old Caucasian woman presented to the out-patient clinic of our department with osteoarthritis of the left hip about six years ago. As conservative treatment had failed, she requested hip replacement arthroplasty. Due to her comparatively younger age and activity level, HRA (Cormet^TM^, Corin Group, Cirencester, UK) was performed. Her post-operative course was unremarkable and the final radiological assessment showed an implant position with a cup inclination of 50° and a stem-shaft angle of 132°. Our patient fully recovered, and the follow-up investigation revealed a stable implant and our patient did not report any pain or problems related to the implant.

**Figure 1:**
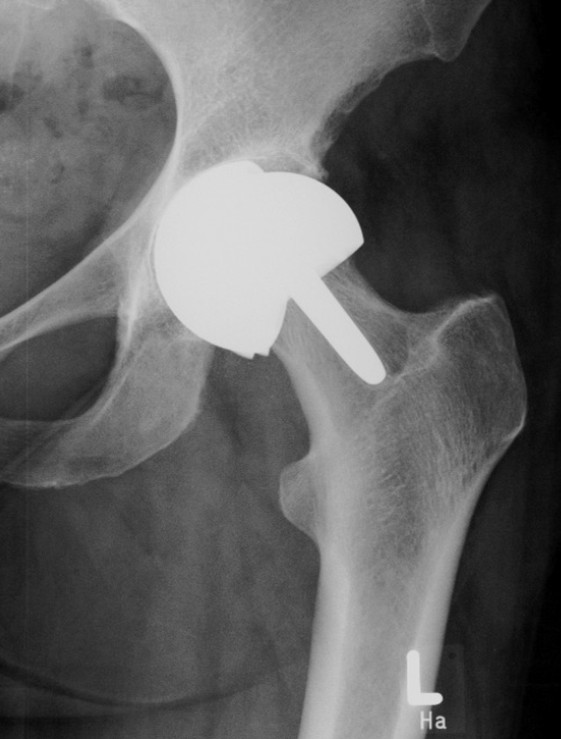
Early failure of hip resurfacing arthroplasty three years after implantation.

**Figure 2:**
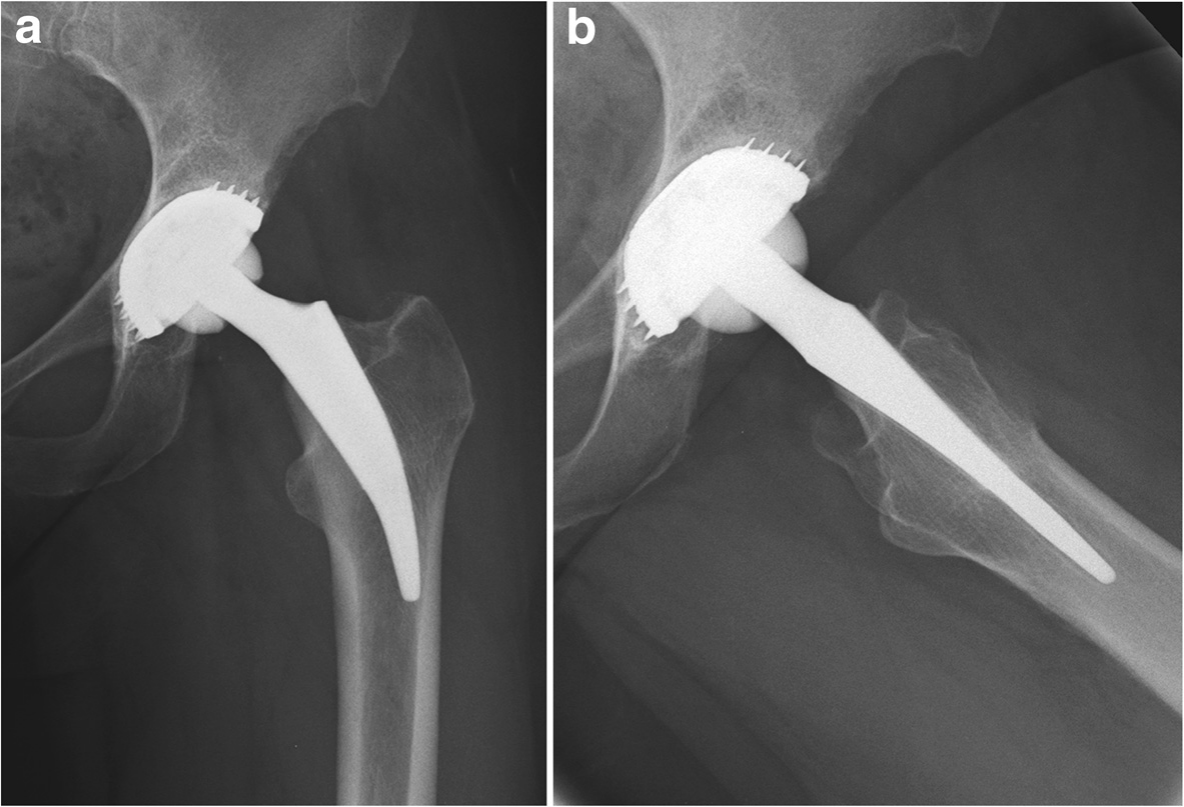
Short-stem hip implant two years after revision surgery in (a) anterior-posterior and (b) lateral views.

Three years later, our patient presented outside the normal follow-up with severe pain in the replaced hip joint. She reported about a falling incident that had occurred two months earlier, followed by an increasing pain over the subsequent weeks. Clinical and radiological evaluation revealed a failure of the acetabular component, which was already dislocated, and additionally showed a narrowing of the femoral neck (Figure 1). For those reasons, our patient underwent revision surgery. Intra-operatively, a massive metallosis of the peri-prosthetic tissue was found and the femoral and acetabular components were already damaged. Therefore removal of the whole implant became necessary. As the femoral bone was found to be intact, osteotomy could be performed directly below the femoral component. By doing this, preservation of the femoral neck was possible, which allowed a revision with a metaphyseal-anchored short-stem hip implant (Metha®, B. Braun AesculapOrthopedics, Tuttlingen, Germany) (Figure 2a,b).

Post-operatively, our patient recovered well and was subsequently referred to a rehabilitation facility. Mobilization was performed by default with half body weight until soft tissue healing was accomplished (two weeks), followed by a rapid and pain-adapted increase to full weight bearing. The follow-up sessions at one, three, six, 12 and 24 months post-operatively were regular. The radiographs at the two-year follow-up showed a stable implant position (Figure 2a,b). Clinical function two years after revision was good, with a Harris Hip Score of 86, a University of California, Los Angeles(UCLA) score of six and a Western Ontario and McMaster Universities Arthritis Index (WOMAC) score of 12.6, with 3.8 in the category ‘pain’, 1.7 in the category ‘stiffness’ and 7.1 in the category ‘function’.

## Discussion

Preservation of bone stock in younger patients requiring hip replacement is important since those patients will most likely experience at least one implant revision during their remaining lifetime [1]. Our patient was provided with HRA, as the implant design has shown good clinical function and dislocation rates as well as high sports activity levels [6,7]. Furthermore, several studies have demonstrated a satisfying mid-term and long-term outcome [6]. However, it has recently become apparent that HRA also compromises the risk of early failure in certain collectives; especially in women with small implants, as seen in our patient [8]. Although we are not able to state what finally caused the early implant failure in our patient’s case, HRA preserved femoral bone stock and thereby facilitated revision surgery. This is of major importance as, beside damage of the soft tissue, bone loss represents one of the main reasons leading to an impaired function after revision surgery [3]. Since many patients with a failed HRA are aged less than 60 years [4,5], it is necessary to devise a long-term strategy.

Up to now, only data have been published that describe revision of HRA with a conventional hip stem [4,5,9]. Moreover, SHA has so far only been used for primary hip replacement [10-12]. Sanguesa-Nebot *et al*. reported the case of a patient with a broken cementless conventional stem that was revised with SHA. As the tip of the implant was broken and stuck in the distal femur, removal would have caused considerable bone and soft tissue damage [13]. Therefore they used a Proxima® short-stem, which is shorter compared to a conventional stem, but has a resection level similar to standard implants and also has a size which, at least at the proximal part, is as large as conventional stems.

In our patient’s case we used a metaphyseally-anchored short-stem design, which preserves clearly more bone stock at the proximal femur, but requires a resection level closely under the femoral head. By doing this, the femoral neck ring is preserved, which is needed for a firm anchorage of the implant. If those prerequisites are met, good primary stability of the SHA implant can be achieved [14].

So far, good functional results and good short-term and mid-term survival rates have been reported for various short-stem hip designs [10-12]. Advantages of SHA include a more physiological load transfer at the metaphyseal part of the femur and a reduced soft tissue trauma, as the small and curved designs facilitate the preparation of the femoral cavity and the insertion of the stem [12]. As a result, faster post-operative mobilization with a reduced hospital stay has been reported [15]. A further advantage of SHA is the preservation of the femoral bone stock. This allows the use of a conventional stem should a revision become necessary, thus avoiding revision implants with an inferior outcome. At the same time, all acetabular cups, bearing surfaces and head sizes that are used for conventional total hip arthroplasty can also be applied for SHA. For those reasons, SHA offers an attractive alternative for younger patients requiring hip replacement and, as shown in this report, can also be used to revise a HRA implant. Regardless, it should be noted that to date, only short-term and mid-term results are available for SHA and these results still have to be confirmed by long-term studies.

## Conclusions

This case report demonstrates that revision of hip resurfacing arthroplasty can not only be performed with a conventional hip implant, but also with a bone-conserving short-stem hip implant. This is of particular importance as it allows further preservation of the femoral bone stock and helps to provide a long-term solution to younger patients with a high risk of further revisions.

## Consent

Written informed consent was obtained from the patient for publication of this case report and any accompanying images. A copy of the written consent is available for review by the Editor-in-Chief of this journal.

## Competing interests

The authors declare that they have no competing interests.

## Authors’ contributions

FS and LWJ wrote the manuscript. FM, AF and VJ performed and planed the surgeries, the post-operative care, the follow-up of our patient and acquired the clinical data. All authors read and approved the final manuscript.
